# Supplementary material for: EnzML: multi-label prediction of enzyme classes using InterPro signatures
Source: BMC Bioinformatics. 2012 Apr 25;13:61. doi: 10.1186/1471-2105-13-61 (PMC3483700; doi:10.1186/1471-2105-13-61)
Supplement: Addtional file 5 — The Java code to format the data files, evaluate and predict. The file enzml_java_code.tar.gz contains the Java code used to format database data to ARFF and XML formats, to execute cross and train-test (jackknife) evaluations and to record evaluation results to database. More information is included in the readme.txt file and the Javadoc files. The code can be used with a MySQL database. To use a different database software, other JDBC drivers might be required. [file 1471-2105-13-61-S5.gz › java_code/enzml2011/doc/uk/ac/ed/inf/taweka/mulan/learn/ExperimentTable.html]

ExperimentTable


---


|  |  |  |
| --- | --- | --- |
| **Overview** \| **Package** \| **Class** \| **Use** \| **Tree** \| **Deprecated** \| **Index** \| **Help** | |  |
| **PREV CLASS**   **NEXT CLASS** | **FRAMES**    **NO FRAMES**     **All Classes** |  |
| SUMMARY: NESTED \| FIELD \| CONSTR \| METHOD | DETAIL: FIELD \| CONSTR \| METHOD |  |


---


## uk.ac.ed.inf.taweka.mulan.learn Class ExperimentTable

```
java.lang.Object
  uk.ac.ed.inf.utils.database.Table
      uk.ac.ed.inf.taweka.mulan.learn.ExperimentTable
```

---

``` public class ExperimentTable extends uk.ac.ed.inf.utils.database.Table ```

Gives the names and table field names for multi-label specific evaluation
measures (such as micro-accuracy, macro-accuracy, hamming loss etc.)

**Version:**
:   8 Jun 2010

**Author:**
:   Luna De Ferrari luna.deferrari-at-ed.ac.uk

---

| **Field Summary** | |
| --- | --- |
| `static java.lang.String` | `ALGORITHM`             the name for the algorithm table column |
| `static java.lang.String` | `END_TIMESTAMP`             time at end of computation |
| `static java.lang.String` | `EXPERIMENTS_TABLE_NAME`             the name for the table to store the evaluation results |
| `static java.lang.String` | `RUN`             the evaluation run number (eg: between 0 and 9 for a 10 fold cross evaluation) |
| `static java.lang.String` | `START_TIMESTAMP`             time at beginning of computation |
| `static java.lang.String` | `TEST_ARFF_ID`             the table column to store the arff file id |
| `static java.lang.String` | `TRAIN_ARFF_ID`             the table column to store the arff file id |


**Constructor Summary**

`ExperimentTable()`


| **Method Summary** | |
| --- | --- |
| `static java.util.Vector<uk.ac.ed.inf.utils.database.TableColumn>` | `getColumnDefinitions()` |
| `static java.util.Hashtable<java.lang.String,java.lang.String>` | `getMeasureFieldNames()`             A hashtable containing for each evaluation measure the corresponding table field name. |
| `static java.util.Hashtable<java.lang.String,uk.ac.ed.inf.utils.database.TableColumn>` | `getMeasureFields()`             A hashtable containing for each measure field name the corresponding table field object (name + sql data type). |
| `static java.lang.String` | `getMeasureTableField(java.lang.String evaluationMeasureName)`             For each evaluation measure, gets the corresponding table field The field name is the evaluation measure name, all lower-case, with spaces and dashes substituted with underscores. |
| `static void` | `main(java.lang.String[] args)` |

**Methods inherited from class uk.ac.ed.inf.utils.database.Table**

`getColumnNames, getColumns, getTableName, hasKey`

**Methods inherited from class java.lang.Object**

`equals, getClass, hashCode, notify, notifyAll, toString, wait, wait, wait`

**Field Detail**

### ALGORITHM

```
public static final java.lang.String ALGORITHM
```

:   the name for the algorithm table column

    **See Also:**: Constant Field Values

---


### TRAIN\_ARFF\_ID

```
public static final java.lang.String TRAIN_ARFF_ID
```

:   the table column to store the arff file id

    **See Also:**: Constant Field Values

---


### TEST\_ARFF\_ID

```
public static final java.lang.String TEST_ARFF_ID
```

:   the table column to store the arff file id

    **See Also:**: Constant Field Values

---


### END\_TIMESTAMP

```
public static final java.lang.String END_TIMESTAMP
```

:   time at end of computation

    **See Also:**: Constant Field Values

---


### EXPERIMENTS\_TABLE\_NAME

```
public static final java.lang.String EXPERIMENTS_TABLE_NAME
```

:   the name for the table to store the evaluation results

    **See Also:**: Constant Field Values

---


### RUN

```
public static final java.lang.String RUN
```

:   the evaluation run number (eg: between 0 and 9 for a 10 fold cross
    evaluation)

    **See Also:**: Constant Field Values

---


### START\_TIMESTAMP

```
public static final java.lang.String START_TIMESTAMP
```

:   time at beginning of computation

    **See Also:**: Constant Field Values


**Constructor Detail**

### ExperimentTable

```
public ExperimentTable()
```


**Method Detail**

### getColumnDefinitions

```
public static java.util.Vector<uk.ac.ed.inf.utils.database.TableColumn> getColumnDefinitions()
```

---


### getMeasureFieldNames

```
public static java.util.Hashtable<java.lang.String,java.lang.String> getMeasureFieldNames()
```

:   A hashtable containing for each evaluation measure the corresponding
    table field name.

    :   **Returns:**: a 'measure name-to-field name' hash table

---


### getMeasureFields

```
public static java.util.Hashtable<java.lang.String,uk.ac.ed.inf.utils.database.TableColumn> getMeasureFields()
```

:   A hashtable containing for each measure field name the corresponding
    table field object (name + sql data type).

    :   **Returns:**: a 'field name-to-field' hash table

---


### getMeasureTableField

```
public static java.lang.String getMeasureTableField(java.lang.String evaluationMeasureName)
```

:   For each evaluation measure, gets the corresponding table field The field
    name is the evaluation measure name, all lower-case, with spaces and
    dashes substituted with underscores.

    :   **Returns:**: a column name taken from a Mulan `Measure` name

---


### main

```
public static void main(java.lang.String[] args)
```


---


|  |  |  |
| --- | --- | --- |
| **Overview** \| **Package** \| **Class** \| **Use** \| **Tree** \| **Deprecated** \| **Index** \| **Help** | |  |
| **PREV CLASS**   **NEXT CLASS** | **FRAMES**    **NO FRAMES**     **All Classes** |  |
| SUMMARY: NESTED \| FIELD \| CONSTR \| METHOD | DETAIL: FIELD \| CONSTR \| METHOD |  |


---
